# Supplementary material for: Adaptation of reach action to a novel force-field is not predicted by acuity of dynamic proprioception in either older or younger adults
Source: Exp Brain Res. 2020 Dec 14;239(2):557–74. doi: 10.1007/s00221-020-05997-3 (PMC7936968; doi:10.1007/s00221-020-05997-3)
Supplement: Supplementary file 1 — Supplementary file1 (PDF 3775 KB) [file 221_2020_5997_MOESM1_ESM.pdf]

Supplementary Material for:

Adaptation of reach action to a novel force-field is not predicted by acuity of dynamic proprioception in either older or younger adults

Authors: Nick M. Kitchen[1,2*], R Chris Miall[1]

[1] School of Psychology, University of Birmingham, Birmingham, UK.

[2] Dept. of Speech & Hearing Science, University of Washington, Seattle, WA, USA.

Email for correspondence: nickkitchen1@gmail.com

1. Extended Analysis of Force-Field Adaptation Kinematics

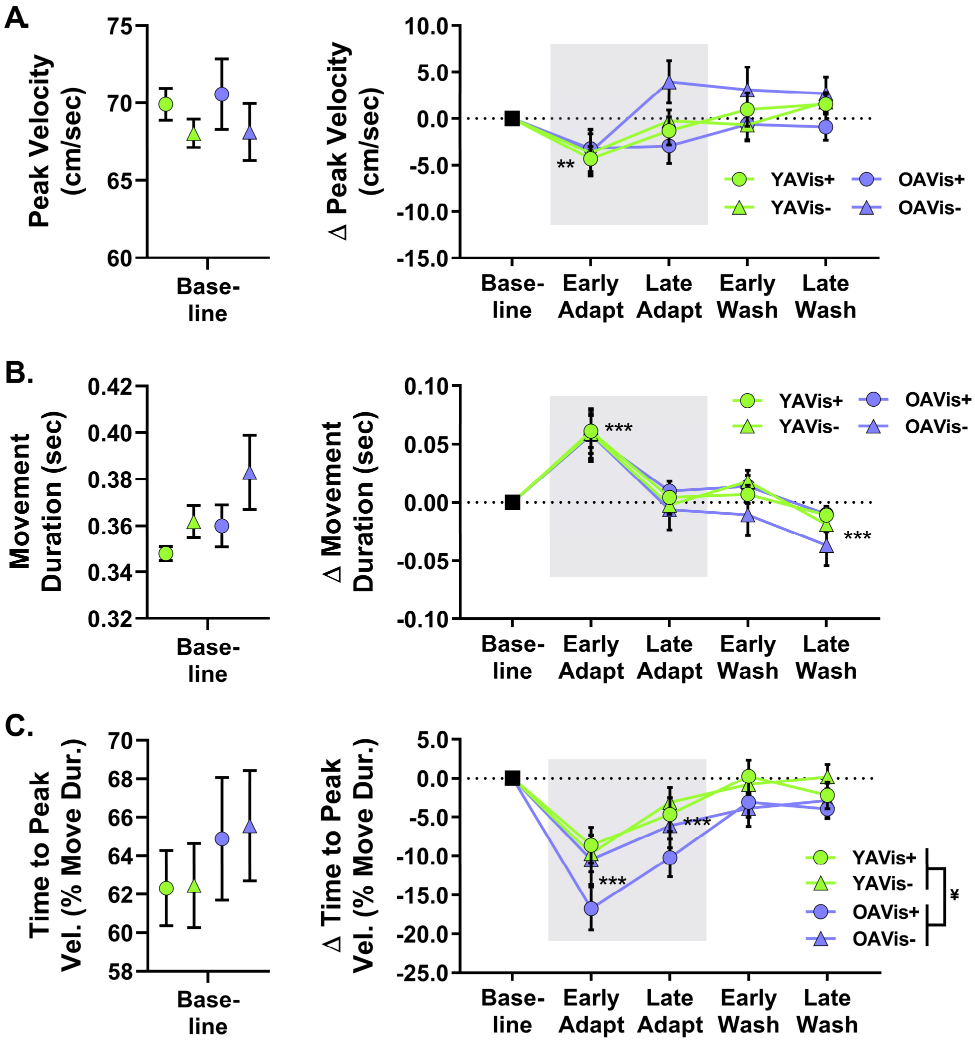

Figure S1. – Group average (± 1SE) kinematic data for the force-field adaptation task across different phases of the experiment. All data is normalized to baseline (late null) performance which is shown in the left of each panel. **A.** shows the peak velocity data, **B.** shows the movement duration and **C.** shows the time to peak velocity represented as a percentage of total movement duration. Significant differences between phases is shown as ****** (p_{adj} < 0.01), ******* (p_{adj} < 0.001) and effect of age group indicated by ¥ (p < 0.05).

Normalized peak velocity – There was an effect of phase on normalised peak velocity ($F[2.5, 152.5] = 10.0$, $p < 0.001$, $\eta^2_p = 0.14$) with follow-up comparisons indicating that peak velocity dropped in early adaptation compared to all other phases (all $t[64] \geq 3.21$, $p_{adj} \leq 0.005$ – Figure S1-A). There were no two- or three-way interactions of phase (all $p > 0.262$), as well as no effects or interactions of vision and age group on normalised peak velocity (all $p > 0.180$).

Normalized movement duration – There was also an effect of phase on normalised movement duration ($F[1.9, 117.1] = 35.6$, $p < 0.001$, $\eta^2_p = 0.36$) where movement duration increased in early adaptation compared to all other phases (all $t[66] \geq 5.40$, $p_{adj} < 0.001$), but was similar from late adaptation to early washout ($t[66] = -1.0$, $p_{adj} = 0.319$), and then finally reduced in late washout compared to all other phases (all $t[66] \geq 3.85$, $p_{adj} < 0.001$ – Figure S1-B). The two- and three-way interactions of phase were not significant (all $p > 0.588$), and there were no effects or interactions of vis and age group (all $p > 0.318$).

Normalized time to peak velocity – There was an effect of phase on normalised time to peak velocity (TPV – $F[2.4, 152.8] = 28.6$, $p < 0.001$, $\eta^2_p = 0.31$), where TPV dropped to its shortest in early adaptation ($t[67] \geq 3.85$, $p_{adj} < 0.001$ compared to all other phases), recovered towards baseline by late adaptation (all $t[67] \geq 3.85$, $p_{adj} < 0.001$ compared to other phases) and then remained stable at near baseline levels during washout (early vs late washout; $t[67] = 0.38$, $p_{adj} = 0.703$ – Figure S1-C). However, there were no two- or three-way phase interactions on normalised TPV (all $p > 0.274$). There was no between-subjects effect of vis group on normalised TPV ($F[1, 64] = 0.87$, $p = 0.354$), however there was an effect of age group on TPV ($F[1, 64] = 4.6$, $p = 0.036$, $\eta^2_p = 0.07$) where overall, older adults reduced their TPV relative to baseline (and hence reached peak velocity earlier in their movements) more than younger adults. The vis group x age group interaction was not significant ($F[1, 64] = 0.43$, $p = 0.511$).

2. Proprioception and Force-Field Adaptation Correlation Tables

BL Uncertainty Range vs. Adaptation

PVLD

Group		Early				Late			
		r	*p*	p_{fdr}	BF_{01}	*r*	*p*	p_{fdr}	BF_{01}
Older	Vis+	0.341	0.196	0.556	1.497	0.113	0.689	0.982	2.918
	Vis-	-0.006	0.982	0.982	3.241	-0.071	0.785	0.982	3.226
Younger	Vis+	-0.355	0.162	0.556	1.346	-0.042	0.879	0.982	3.207
	Vis-	-0.086	0.742	0.982	3.174	-0.332	0.209	0.556	1.562

Adaptation Index

Group		Early				Late			
		r	*p*	p_{fdr}	BF_{01}	*r*	*p*	p_{fdr}	BF_{01}
Older	Vis+	-0.363	0.167	0.462	1.341	-0.473	0.075	0.462	0.734
	Vis-	0.026	0.920	0.920	3.323	0.217	0.404	0.462	2.416
Younger	Vis+	0.266	0.319	0.462	2.070	0.296	0.249	0.462	1.802
	Vis-	0.230	0.375	0.462	2.317	0.322	0.224	0.462	1.639

Table S1. – Group correlations between baseline (BL) uncertainty range and performance early and late adaptation block, as measured separately by peak-velocity lateral deviation (PVLD) and adaptation index. Correlation coefficients (*r*) are provided alongside raw (*p*) and false discovery rate-adjusted (p_{fdr}) *p*-values. Bayes factor for correlation indicates increasing likelihood for null as BF_{01} increases from 1 and increasing likelihood for alternative as BF_{01} decreases from 1 (see main text for further details).

BL Absolute Bias vs. Adaptation

PVLD

Group		Early				Late			
		r	*p*	p_{fdr}	BF_{01}	*r*	*p*	p_{fdr}	BF_{01}
Older	Vis+	-0.131	0.616	0.821	2.970	-0.202	0.454	0.726	2.500
	Vis-	0.299	0.261	0.726	1.808	0.443	0.075	0.598	0.767
Younger	Vis+	-0.199	0.444	0.726	2.545	-0.044	0.871	0.918	3.202
	Vis-	0.277	0.282	0.726	1.951	0.027	0.918	0.918	3.322

Adaptation Index

Group		Early				Late			
		r	*p*	p_{fdr}	BF_{01}	*r*	*p*	p_{fdr}	BF_{01}
Older	Vis+	-0.017	0.949	0.999	3.332	-0.191	0.478	0.765	2.568
	Vis-	-0.272	0.290	0.581	1.988	-0.478	0.052	0.211	0.586
Younger	Vis+	0.492	0.053	0.211	0.575	< 0.001	0.999	0.999	3.336
	Vis-	-0.321	0.209	0.556	1.604	-0.146	0.576	0.767	2.886

Table S2. – Group correlations between baseline (BL) absolute bias and performance early and late adaptation block, measured separately by peak-velocity lateral deviation (PVLD) and adaptation index. Correlation coefficients (r) are provided alongside raw (*p*) and false discovery rate-adjusted (p_{fdr}) *p*-values. Bayes factor for correlation indicates increasing likelihood for null as BF_{01} increases from 1 and increasing likelihood for alternative as BF_{01} decreases from 1 (see main text for further details).

Adaptation Extent vs. Bias Shift

Group		PVLD			
		r	*p*	p_{fdr}	BF_{01}
Older	Vis+	0.255	0.340	0.679	2.127
	Vis-	0.386	0.126	0.505	1.131
Younger	Vis+	0.009	0.973	0.998	3.240
	Vis-	0.001	0.998	0.998	3.339

Group		Adaptation Index			
		r	*p*	p_{fdr}	BF_{01}
Older	Vis+	-0.354	0.163	0.326	1.355
	Vis-	0.462	0.071	0.285	0.724
Younger	Vis+	-0.142	0.600	0.661	2.854
	Vis-	0.115	0.661	0.661	3.054

Table S3. – Group correlations between adaptation extent and proprioceptive recalibration (bias shift) across the adaptation block. Adaptation extent is measured separately by peak-velocity lateral deviation (PVLD) and adaptation index. Correlation coefficients (r) are provided alongside raw (*p*) and false discovery rate-adjusted (p_{fdr}) *p*-values. Bayes factor for correlation indicates increasing likelihood for null as BF_{01} increases from 1 and increasing likelihood for alternative as BF_{01} decreases from 1 (see main text for further details). In all cases, positive correlations indicate greater adaptation was associated with greater shifts in the direction of the perturbing force-field.

3. Age Effects and Correlations of Spatial Working Memory Capacity

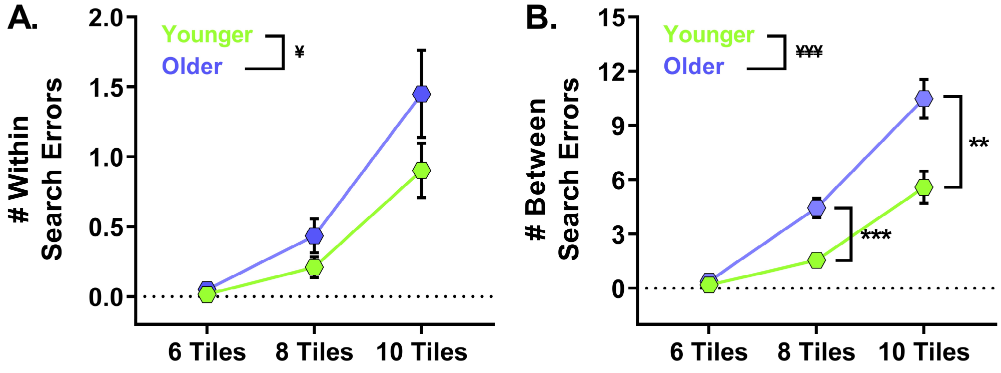

Figure S2. – Group average (± 1SE) search errors for older (purple) and younger (green) adults in the spatial working memory task, where task difficulty increased with number of tiles. **A.** Number of within search errors, where main effect of age is indicated by ¥ ($p < 0.05$). **B.** Between search errors, where main effect of age is indicated by ¥¥¥ ($p < 0.001$) and significant post-hoc comparisons of older and younger adults are indicated by ** ($p_{adj} < 0.01$) and *** ($p_{adj} < 0.001$).

Total SWM Search Errors vs. Adaptation

PVLD

Group		Early				Late			
		r	*p*	p_{fdr}	BF_{01}	*r*	*p*	p_{fdr}	BF_{01}
Older	Vis+	-0.094	0.721	0.940	3.146	0.201	0.456	0.730	2.509
	Vis-	-0.232	0.386	0.730	2.292	0.032	0.902	0.940	3.315
Younger	Vis+	0.460	0.063	0.384	0.678	0.260	0.331	0.730	2.095
	Vis-	0.417	0.096	0.384	0.925	0.020	0.940	0.940	3.330

Adaptation Index

Group		Early				Late			
		r	*p*	p_{fdr}	BF_{01}	*r*	*p*	p_{fdr}	BF_{01}
Older	Vis+	-0.038	0.885	0.885	3.306	-0.074	0.784	0.885	3.131
	Vis-	0.076	0.772	0.885	3.211	0.211	0.417	0.885	2.458
Younger	Vis+	-0.345	0.191	0.763	1.470	-0.494¥	0.044	0.350	0.509
	Vis-	-0.102	0.696	0.885	3.111	0.159	0.542	0.885	2.806

Table S4. – Group correlations between total search errors in the spatial working memory (SWM) task and early and late performance in the adaptation block, measured separately by peak-velocity lateral deviation (PVLD) and adaptation index. Correlation coefficients (r) are provided alongside raw (*p*) and false discovery rate-adjusted (p_{fdr}) *p*-values. Significant correlations that did not survive corrections for multiple comparisons indicated by ¥. Bayes factor for correlation indicates increasing likelihood for null as BF_{01} increases from 1 and increasing likelihood for alternative as BF_{01} decreases from 1 (see main text for further details).

Total SWM Search Errors vs. Washout

PVLD

Group		Early				Late			
		r	*p*	p_{fdr}	BF_{01}	*r*	*p*	p_{fdr}	BF_{01}
Older	Vis+	-0.196	0.452	0.755	2.567	-0.265	0.303	0.755	2.042
	Vis-	-0.066	0.802	0.917	3.243	-0.155	0.566	0.755	2.785
Younger	Vis+	0.016	0.950	0.950	3.332	-0.180	0.489	0.755	2.672
	Vis-	-0.333	0.192	0.755	1.516	-0.170	0.500	0.755	2.778

Adaptation Index

Group		Early				Late			
		r	*p*	p_{fdr}	BF_{01}	*r*	*p*	p_{fdr}	BF_{01}
Older	Vis+	-0.132	0.614	0.702	2.966	0.274	0.286	0.573	1.971
	Vis-	0.366	0.148	0.573	1.269	0.170	0.544	0.702	2.651
Younger	Vis+	-0.283	0.271	0.573	1.903	0.014	0.957	0.957	3.334
	Vis-	0.492[¥]	0.045	0.360	0.520	0.222	0.392	0.627	2.375

Table S5. – Group correlations between total search errors in the spatial working memory (SWM) task and early and late performance in the washout block, measured separately by peak-velocity lateral deviation (PVLD) and adaptation index. Correlation coefficients (r) are provided alongside raw (*p*) and false discovery rate-adjusted (p_{fdr}) *p*-values. Significant correlations that did not survive corrections for multiple comparisons indicated by [¥]. Bayes factor for correlation indicates increasing likelihood for null as BF_{01} increases from 1 and increasing likelihood for alternative as BF_{01} decreases from 1 (see main text for further details).
